# Creating a web-based digital photographic archive: one hospital library’s experience

**DOI:** 10.5195/jmla.2017.220

**Published:** 2017-04

**Authors:** Caroline Marshall, Janet Hobbs

## Abstract

**Background:**

Cedars-Sinai Medical Center is a nonprofit community hospital based in Los Angeles. Its history spans over 100 years, and its growth and development from the merging of 2 Jewish hospitals, Mount Sinai and Cedars of Lebanon, is also part of the history of Los Angeles. The medical library collects and maintains the hospital’s photographic archive, to which retiring physicians, nurses, and an active Community Relations Department have donated photographs over the years. The collection was growing rapidly, it was impossible to display all the materials, and much of the collection was inaccessible to patrons.

**Case Presentation:**

The authors decided to make the photographic collection more accessible to medical staff and researchers by purchasing a web-based digital archival package, Omeka. We decided what material should be digitized by analyzing archival reference requests and considering the institution’s plan to create a Timeline Wall documenting and celebrating the history of Cedars-Sinai.

**Conclusion:**

Within 8 months, we digitized and indexed over 500 photographs. The digital archive now allows patrons and researchers to access the history of the hospital and enables the library to process archival references more efficiently.

## INTRODUCTION

As information rapidly accumulates and multiplies, many libraries become depositories, storing large quantities of papers, photographs, and other items in boxes on shelves. Subsequent retrieval of these items can be problematic and unappealing to those searching for information. This scenario is why certain library collections can be perfect candidates for digitization [1]. Although an increasing number of health sciences libraries are digitizing collections—whether for scholarly output, historical documentation, or research—there is little literature on digital collection content, accessibility, or standards [2, 3].

The purpose of digitizing information is to make materials discoverable, to create flexibility so that the materials can be accessed from anywhere at any time, and to allow materials to be easily interrogated and analyzed [4]. In the interest of preservation and dissemination, libraries are increasingly digitizing their collections [5]. High-quality digitization can provide the researcher with enough information so that there is no need to handle the original material [2], and instead of travelling to the archive, the archive comes to the researcher. Items can be examined by researchers and shared with others both nationally and internationally. Digital communities can be created, offering opportunities for sharing content and collaborating.

Preserving institutional materials is important for documenting the work of an institution and its goals, values, and activities, helping understanding of its history and role in the community [6]. A hospital archival collection describes how the hospital performs and provides medical care to its patients and the community [7]. The focus of health care is the organization, advancement, and delivery of patient care, and there is little time for collecting and preserving materials that can document a hospital’s growth and change [8]. Creating a digital archive is one way a library can add value to the organization by systematically collecting and preserving materials, which can serve to raise the library’s profile [9, 10]. Medical libraries with historical collections need to “seize the initiative” to digitize their archives before the archives become irrelevant [11].

## STUDY PURPOSE

The hospital’s photographic archive collection is managed by the medical library. The collection details over 100 years of the institution’s history. The images chart the history of the 2 hospitals, Cedars of Lebanon and Mount Sinai, that merged to form the Cedars-Sinai Medical Center and describe the growth and development of the medical center and its role in the Los Angeles community. Over the years, our Community Relations Department and retiring physicians and medical staff have donated photographs. The library staff store, catalog, and retrieve the historic photographs for different departmental and hospital events. Unfortunately, these photographs could not be viewed or accessed easily by researchers, staff, or librarians. We had no way of displaying our collection in an easy-to-use format that would allow patrons to search the collection and locate and retrieve images.

We decided to digitize the collection and select a software package that would allow the collection to be displayed, easily accessed, and searched. An interactive archival collection would enable patrons and researchers to search for and download photographs. Our needs were simple: a photographic digital display database that was accessible, easy to use, budget friendly, and searchable to allow patron interaction but with a main focus on display. Furthermore, an interactive database would allow users to review photographs for research or promotional activities in the hospital without having to touch the original photos.

## CASE PRESENTATION

Our collection of photographs comprised images of the founding hospitals that merged to create Cedars-Sinai, staff, physicians, new buildings, services, and notable events such as groundbreakings, award ceremonies, and fundraisers from 1902 to the 1980s. The black-and-white photographs were stored in approximately seventeen boxes and in a number of folders in several filing cabinets. Although a finding aid had been created, it did not cover the whole collection, and there were still several boxes with unknown contents. On average, we received about fifty-five to sixty requests for photographs annually, with many of the requests for multiple photographs. When requests were received, pertinent folders were searched by a librarian, and a selection of photographs was either scanned or given to the requestors so that they could choose and scan the appropriate photographs. This process was time consuming and often led to original photographs being handled many times.

In the fall of 2013, we looked into ways of displaying our large archival photograph collection and making it more accessible to patrons and researchers outside the hospital. We initially decided that a Microsoft Access database with links to scanned photographs would serve our archival needs. However, it soon became clear that staff did not have enough expertise to create such a database or to interface it with the web to make it accessible both inside and outside the institution. Instead, we chose Omeka, a free open source content management system developed by the Roy Rosenweig Center for History and New Media at George Mason University in Fairfax, Virginia. Omeka is easy to use, is budget friendly, and has good search capabilities. Themed exhibitions can also be created from the photographs. We did not pursue other packages as we felt Omeka met our needs. After a demonstration of Omeka to staff members, we purchased a storage plan on an Omeka server that supported our current needs. As we had a small staff who were fully occupied with their own responsibilities, additional staff members were required to scan photographs and enter the metadata. Two interns were recruited from the Information and Library Science Departments at the University of California, Los Angeles, and San Jose State University.

Selecting photographs to be digitized can be an arduous task. We have a collection policy for the archive; however, because of the large amount of material that had accumulated over the years, we had to be very specific about what we wanted to digitize. We needed a clear idea of what should be digitized and how we should evaluate materials in terms of their historical importance. Our aim was to both visually signpost the development of Cedars-Sinai and to ensure a collection of digitized photographs of potential value to a researcher. A number of questions can be asked to provide insight into the selection process [4]:

- What is the intellectual quality of the material to be digitized?
- Will digitization enhance the value of the source material by not only making it discoverable, but also maintaining or enhancing the resolution quality so that a researcher does not need to touch or examine the original photograph?
- Will digitization make the materials more accessible so that usage is increased and new audiences can be reached?
- Furthermore, the archivist must be familiar with the organization’s history, including key players and milestones, so that they can develop a balanced archive that anticipates what future researchers may want to see [12].

To start the selection process, we analyzed archival reference questions from the previous two years, the majority of which were requests for photographs. The materials were requested by the Marketing or Community Relations Departments and other departments and individuals. The Marketing and Community Relation Departments’ requests were often for photographs to use in memorials for deceased physicians, celebrations for retiring directors, or historical pieces on prominent physicians such as Jeremy Swan and William Ganz, doctors who developed a pulmonary artery catheter to monitor heart function. We also looked at the plans for the Timeline Wall, which the institution was creating to highlight the history of Cedars-Sinai Medical Center, to determine which photographs from the archive would be used. Before we started scanning photographs, we addressed copyright issues. Because these photographs would be in the public domain as opposed to behind the institution firewall, we sent our plans to our legal department to ensure we were not breaching copyright laws.

We started by digitizing photographs of former prominent physicians and staff, many of who had been involved in committees to raise funds for constructing new buildings, and other key people, such as the chairs of the Board of Directors who had charted the hospital’s course. We then selected photographs of buildings, building plans, and groundbreakings for building expansions to show how the hospital had grown through the years. We digitized 500 photographs, uploaded them to Omeka, and added metadata.

Digitization standards and guidelines vary from project to project. As there is no overall model or best practice, individual libraries create their own standards [13]. We scanned black-and-white photographs on our current Epson scanner as JPEG files at a resolution of 600 dots per inch (dpi), which was the scanner’s highest quality resolution. Although a 600-dpi scan creates a larger file, it ensures that details are recorded. We scanned in grayscale because scanning the photographs in black-and-white produced an extreme contrast between the 2 colors. Although scanning the photographs as TIFF files would have provided higher-quality digital images with the least amount of compression and, therefore, the lowest loss of image data, they take a long time to load, which can hinder usage. By contrast, JPEG files can be selectively compressed to make smaller files. Although this leads to a loss in image data, it provides a good tradeoff between image quality and storage. JPEGs are also a good choice for image use on the web, as they can be opened and loaded quickly. Originally, we decided to scan the photographs as JPEG files and TIFF files and store the TIFF files on CDs to provide better quality printed photographs for patrons’ needs. However, the TIFF files took up too much storage space, so we scanned as JPEG files only.

We wanted the photographs to be searchable from a number of access points, but the data needed to be entered in a consistent manner. Dublin Core is a commonly used metadata standard that provides eighteen descriptors that can be used to search data. Although Omeka supports Dublin Core, it does not provide prompts or error messages if the data are entered incorrectly. Therefore, the interns created a template on paper for entering data using Dublin Core. The template comprised directions and examples of how data should be entered into each field and what punctuation should be used to ensure uniformity across the collections. As each group of photographs was scanned and then uploaded into Omeka, metadata were added such as title, description, date, and subject headings. We did not use all Dublin Core descriptors. The original photographs were labeled and stored with their Omeka item numbers. The original scans were deleted at a later date to conserve storage. In some cases, the collection contained up to eight copies of an individual photograph; in these cases, we scanned one copy and kept two copies of the originals. A workflow chart was developed to organize scanning, storing, and uploading photographs. A part-time staff member, a former cataloger, oversaw the selection of subject headings.

In the fiscal year 2015/16, the digital archive had been visited 4,000 times. The accessibility of the photographic archive has allowed other departments to access our historical photographs. Most recently, the Department of Neurology created a History of Neurology at Cedars-Sinai. Their request for photographs of historical buildings was answered by quickly demonstrating how to use the digital archive, which allowed them to browse the photographs in our Hospital Building Collection and select from a variety of images. We continue to work with the Community Relations Department and will collaborate further when they extend the Timeline Wall. We recently provided photographs for a physician’s presentation on the History of Medicine. We are also working with the Department of Nursing to collect and digitize photographic materials that describe their professional development through the years.

## DISCUSSION

Our digital image archive was completed within 8 months and was made public at the same time as the Timeline Wall for maximum exposure. The library was fortunate that the Timeline Wall was created in the same corridor as the library and crossed the library doors. Visitors who entered the library from viewing the Timeline Wall were shown the digital archive and how to access it. We also published a piece in the company newsletter and placed the link to the archive on our web page. Several months later, we decided to upgrade our Omeka plan to allow us to continue the digitization project, and more than 300 photographs were added. We are still uncovering photographs as we process other materials stored in the library.

One lesson we learned was that a location field would have been useful to indicate where the original photographs were stored. To avoid confusion, we decided to store all the photographs of people in one place, and photographs of buildings were stored by building name. All photographs were labeled with their Omeka number, title, and date, if known.

Reviewing the photographs and choosing which ones to scan provided insight into our collection. A number of photographs of people and events were unidentified, and there were often numerous copies of the same photograph. This made us question why we were accepting so many copies and in some cases damaged photographs. As a result, we are working toward a more curated archive.

When we started our archive, we scanned several photographs that in hindsight might not have contributed to the goal of documenting the history of the institution. In our initial push to preserve, we did not always ask ourselves if the material was worth preserving and if it would be useful for research. We scanned several photographs for which we only knew the name of the subject, and further detailed research would be needed to learn more. The question is whether it is worth spending that amount of time on something that may have no value. As we have become more experienced, we are making better-informed decisions. This experience and the knowledge we have gained during the process mirrors similar learning curves in other institutions [8, 14].

We will continue to build the collection and work with library science students. At the same time, we may also consider adding different types of materials, such as videos and oral interviews. Larger items, such as medical instruments, are being photographed and entered into Omeka, as some of these instruments are too big to be displayed and might remain in storage. We will also need to create a strategy for electronic preservation in line with technical trends in digitization to ensure that our materials will remain relevant into the future [14].

There still remains a great deal to do, and we continue to receive material, but as we now have firmer guidelines, the process of creating a digital archive is less daunting. While our project provides a model for other health care institutions, each collection is unique and each library will face its own specific challenges [8]. There is no one standard way for individual institutions to create a digital archive [2], but our experience provides a practical model that other hospitals interested in preserving and engaging in their archival collections can follow.

The Cedars-Sinai Medical Library Omeka site can be seen at cedarssinai.omeka.net. A list of institutions that use Omeka can be seen at www.omeka.org.
